# Could Behaviour and Not Physiological Thermal Tolerance Determine Winter Survival of Aphids in Cereal Fields?

**DOI:** 10.1371/journal.pone.0114982

**Published:** 2014-12-09

**Authors:** Lucy Alford, Thiago Oliveira Andrade, Romain Georges, Françoise Burel, Joan van Baaren

**Affiliations:** UMR 6553 ECOBIO, Université de Rennes I, Rennes Cedex, France; Swedish University of Agricultural Sciences, Sweden

## Abstract

Traits of physiological thermotolerance are commonly measured in the laboratory as predictors of the field success of ectotherms at unfavourable temperatures (e.g. during harsh winters, heatwaves, or under conditions of predicted global warming). Due to being more complicated to measure, behavioural thermoregulation is less commonly studied, although both physiology and behaviour interact to explain the survival of ectotherms. The aphids *Metopolophium dirhodum*, *Rhopalosiphum padi* and *Sitobion avenae* are commercially important pests of temperate cereal crops. Although coexisting, these species markedly differ in winter success, with *R. padi* being the most abundant species during cold winters, followed by *S. avenae* and lastly *M. dirhodum*. To better understand the thermal physiology and behavioural factors contributing to differential winter success, the lethal temperature (physiological thermotolerance) and the behaviour of aphids in a declining temperature regime (behavioural thermotolerance) of these three species were investigated. Physiological thermotolerance significantly differed between the three species, with *R. padi* consistently the least cold tolerant and *S. avenae* the most cold tolerant. However, although the least cold tolerant of the study species, significantly more *R. padi* remained attached to the host plant at extreme sub-zero temperatures than *S. avenae* and *M. dirhodum*. Given the success of anholocyclic *R. padi* in harsh winters compared to its anholocyclic counterparts, this study illustrates that behavioural differences could be more important than physiological thermotolerance in explaining resistance to extreme temperatures. Furthermore it highlights that there is a danger to studying physiological thermotolerance in isolation when ascertaining risks of ectotherm invasions, the establishment potential of exotic species in glasshouses, or predicting species impacts under climate change scenarios.

## Introduction

Due to a limited ability to regulate body temperature above or below ambient, ectotherms are greatly affected by environmental thermal conditions. The survival of ectotherms at unfavourable thermal conditions is governed by their intrinsic physiological thermotolerance, which can be enhanced by behavioural responses. Subsequently, the physiological thermotolerance of ectotherms has received much research attention with laboratory based measures of intrinsic thermal tolerance e.g. lethal temperature [1]–[4], lethal time [5], [6], and supercooling point [7]–[10], commonly used as predictors of the field success of ectotherms at unfavourable temperatures (e.g. during harsh winters, heatwaves, or under conditions of predicted global warming) [11].

However, although a strong link between extreme temperatures and ectotherm survival exists, the relationship between temperature lethality and survival in the field has often yet to be empirically established [2]. Furthermore, when subjected to increasingly low or high temperatures, a suite of behavioural and physiological responses first take place before the point of temperature induced lethality is reached [12], with detrimental effects to fitness occurring as soon as temperatures are reached that impede movement in search of food, a mate, or in escape of predators [13], [14]. As such, the use of non-lethal behavioural thresholds [15], for example, locomotor thresholds [2], [14], [16], [17], critical temperatures [11], [18]–[22], chill coma temperatures [12]; [23]–[25] and chill coma recovery [26]–[28] may be of more importance as they provide more ecologically relevant information [27]. Consequently, such measures have received increased research attention in recent years, particularly within the field of insect thermal biology in regard to enhancing knowledge on the consequences of predicted climate change on insect fitness, abundance and distribution, and the implications for pest control [11], [14], [16], [23], [29]–[31].

While the employment of behavioural measures has undoubtedly increased, much to the benefit of our understanding of the impacts of temperature on ectotherms, measures of thermotolerance are commonly performed under laboratory conditions with little relation to the natural environment of the study species. Consequently, such studies therefore provide little information as to how the species may interact with its environment as a form of behavioural thermoregulation to enhance thermal tolerance or survival at extreme temperatures. With recent research suggesting that ectotherms may not have the physiological thermal safety margins as previously thought, ectotherms will be forced to rely increasingly on behavioural thermoregulation in the face of climate change to avoid and survive unfavourable thermal conditions [32]. As such, there is increasing need for investigation into behavioural thermoregulation, in conjunction with physiological thermal tolerance studies, to fully understand the vulnerability of ectotherms to a changing climate and extreme weather events.

The present study aims to combine measures of physiological thermotolerance with behavioural thermotolerance to better understand low temperature survival, employing aphids as a model. Aphids are pests to many commercially important crops [33], [34]. During winter months, to maximise survival, aphids commonly overwinter as an egg (holocyclic); the most cold tolerant stage of the aphid life cycle [35]. However, where winter conditions permit, in particular in Western Europe, overwintering may occur as anholocyclic individuals which remain active on host plants [36]. It is this ability to continue reproducing during mild winters which greatly increases the potential for aphid population growth, and in turn the potential for, and severity of, spring pest outbreaks [37]–[40]. Given their importance as pest species, much research has therefore focused on the thermal tolerance of aphids to enhance understanding of population dynamics, pest outbreaks, and, in turn, to better inform biological control practices [2], [3], [14], [41]–[46].

The focus aphid species of the current study, *Metopolophium dirhodum* (Walker), *Rhopalosiphum padi* (Linnaeus) and *Sitobion avenae* (Fabricius) are three major pests of commercially important cereal crops throughout temperate climates [33], [47]. Although coexisting, these species markedly differ in their winter success, with *R. padi* being the most abundant species in December and January, followed by *S. avenae* [48]–[50]. *M. dirhodum* has, in recent warmer winters, increased in abundance, but generally remains below that of *S. avenae* [48]. By late winter and early spring, *R. padi* numbers decline and *S. avenae* increases to dominance, creating a new population composition, indicative of a spring aphid community [48]. Here, we aim to link thermal physiology and behaviour to the winter field success of aphids in north-western France. We report on laboratory experiments designed to investigate the thermal physiological and behavioural characteristics of the three aphid species to better understand factors underlying differential winter success.

## Materials and Methods

### Aphid winter abundance

Aphid winter abundance (covering the period of late November to the end of February) was recorded in winter wheat, oat and triticale fields over four consecutive winters (2009/10, 2010/11, 2011/12 and 2012/13) in the Long Term Ecological Research (LTER) site Armorique (http://osur.univ-rennes1.fr/za-armorique/) located in Brittany, north-western France. In early winter, the use of chemical treatments results in limited aphid colonisation to the study fields. As a consequence, the number of fields available for aphid sampling was limited and a total of one field was sampled in winter 2009/10 and two fields in winters 2010/11, 2011/12 and 2012/13. Study fields were sampled on a weekly or fortnightly basis, weather dependent, and aphid abundance was taken as the number of live aphids (nymphs and adults) and aphid mummies of *M. dirhodum*, *R. padi* and *S. avenae* collected in a 40 minute sampling period by two people. Sampling was performed by thoroughly examining the maximum number of plants randomly located in the field during the sampling period. Meteorological data for the winter sampling seasons were recorded at two weather stations of Météo France located at Rennes-Saint Jacques (48°11, −1°67) and Dinard-Pleurtuit (48°63, −2°05). The mean daily minimum and maximum temperature was then determined for one week prior to each sampling period since it has been shown to significantly explain aphid abundances [48].

### Aphid rearing

Stock cultures of anholocyclic *M. dirhodum*, *R. padi* and *S*. *avenae* were established using aphids originally collected in the LTER site Armorique. Aphids were reared on winter wheat, *Triticum aestivum*, ‘Renan’ cultivar grown in vermiculite within Plexiglas cages (50×50×50 cm) and housed in a controlled environmental room at 20±1°C and LD 16∶8 h photoperiod.

### Determination of lower lethal temperature (LT_50_)

Lethal temperature_50_ (i.e. the temperature that results in 50% mortality of the test population) was determined for first instar nymphs and apterous adults of the three aphid species, synchronised in age to within 24 h. Lethal temperature was determined for the two distinct life stages because it is documented that physiological thermotolerance can vary throughout the lifecycle of an aphid [45]. A ‘direct plunge’ protocol was employed [4], [51]. For this, aphids were directly exposed to a range of low temperatures (in the region 0 to −12°C at 1°C intervals) for a duration of 2 h. The range of exposure temperatures was tailored to each species and life stage to incorporate 0 to 100% mortality of test individuals. For each exposure temperature, 50 first-instar nymphs or adults were placed within 0.5 ml Eppendorf tubes at densities of ten individuals per tube. The Eppendorf tubes were then placed individually within a glass boiling tube and the boiling tubes sealed with a sponge stopper to limit air circulation and maintain a stable internal environment. The boiling tubes were held within a test tube holder and lowered into the alcohol bath (Haake F3, Thermo Electron Corp., Karlsruhe, Baden-Württemberg, Germany) set to the desired sub-zero temperature. A thermocouple was placed within an empty Eppendorf tube, set up in the same way as the tubes containing the aphids, enabling the accurate monitoring of the temperature that the aphids experienced.

Following the 2 h exposure period, test aphids were transferred to recovery tubes to recover at the culture temperature of 20°C. Recovery tubes were constructed from blades of *T. aestivum* placed within small glass boiling tubes (approximately 10 mm in diameter). Cotton wool soaked in water was placed in the bottom of each tube to keep the *T. aestivum* fresh for the duration of aphid recovery. The cotton wool was then covered in a layer of fine sand to prevent aphids coming into contact with the water, and the tubes sealed with fine netting. Survival was assessed 48 h after exposure. The procedure was repeated for each exposure temperature.

A handling control was set up on each day of experiments, as detailed above, with the exception that the aphids remained at 20°C for the duration of the exposure period.

### Determination of aphid behaviour in a declining temperature regime

Aphid behaviour in a declining temperature regime was measured for pre-reproductive apterous adults using a glass column connected to a programmable alcohol bath [21]. Preliminary experiments revealed that the experimental set-up was not suitable for experimentation on first-instar nymphs due to their small size. First-instar nymphs were therefore excluded from experiments.

To obtain plant material, individual grains of winter wheat where sown in plant pots comprised of plastic cylinders (9 cm in height and 2.5 cm in diameter) containing moist vermiculite. The diameter of the plant pots was chosen because it allowed the pots to be inserted into the glass column, thus enabling the use of live plant material in experiments as opposed to excised material. Wheat was selected for experiments following 6–9 days of growth at 20°C when the wheat blades measured approximately 7 cm in height.

In all experiments, 5 adult aphids were transferred onto a single blade of wheat using a fine paintbrush. The aphids were allowed to settle onto the wheat blade during a 30 min acclimatization period. The plant pot containing the single wheat blade and test aphids was subsequently inserted into the bottom of the glass column pre-set to the culture temperature of 20°C and the glass column sealed with a sponge stopper to reduce air flow and maintain a stable thermal environment within the inner column. The programmable alcohol bath was set to decrease the temperature of the column from 20°C to −10°C at a rate of 0.75°C min^-1^. The rate of 0.75°C min^-1^ was chosen to prevent inducing a rapid cold hardening response in the test aphids [52], [53].

During the cooling regime, behavioural responses and interactions with the host plant were noted, along with the current temperature within the glass column. Temperature was recorded manually from the thermocouple display reading to an accuracy of 0.1°C. Specific behaviours that were noted included any active movement on or from the host plant, if the aphid fell from the plant as a result of entering a cold-induced torpor (chill coma), or if the aphid remained attached to the host plant following completion of the declining temperature regime i.e. at temperatures of −10°C.

### Statistical analysis

The temperature resulting in 50% mortality of the experimental populations at low temperature exposures (the LT_50_) was determined using Probit analysis in MINITAB, version 16 (Minitab Inc., State College, Pennsylvania). Handling controls resulted in 99–100% survival across all treatments. The natural response rate was therefore assumed to be close to zero and not included in the model [2]. Significant differences in mortality were identified by non-overlapping 95% fiducial limits [54], [55].

For analysis of aphid behaviour in a declining temperature regime, behavioural observations for each species were first assigned to one of three categories: 1) the aphid actively left the host plant; 2) the aphid fell from the host plant as a result of chill coma; 3) the aphid remained attached to the host plant until −10°C was reached. The categorical data were then analysed using a chi-square test to determine if the proportion of individuals exhibiting each behaviour type differed between the three species. The analysis was performed in MINITAB 16 and individual comparisons made using Bonferroni adjustment.

Of the three categories of aphid behaviour, categories 1 (the aphid actively left the host plant) and 2 (the aphid fell from the host plant as a result of chill coma) were further analysed to investigate differences in the temperature at which these behaviours occurred. Distribution fitting analysis was first performed using MINITAB 16 for each species group to determine which distribution best described the data. Data were first converted from a value of temperature to duration of exposure to make data suitable for the distribution fitting analysis. Parametric distribution analysis was subsequently performed using the appropriate distribution, in all cases Normal, to allow for comparison of scale and location parameters and individual comparisons made using Bonferroni 95% confidence intervals [2], [23].

## Results

### Aphid winter abundance

Meteorological data collected from field stations in the Brittany region of north-western France (Rennes: 48°11,-1°67; Dinard: 48°63,-2°05) revealed winter daily minimum temperatures for the study period to average 4.8°C (2009/10), 4.8°C (2010/11), 6.1°C (2011/12), and 5.1°C (2012/13). In addition, the number of days in which temperatures dropped below 0°C totalled 36 in the winter of 2009/10, 29 in 2010/11, 17 in 2011/12 and 24 in 2012/13.

**Figure 1:**
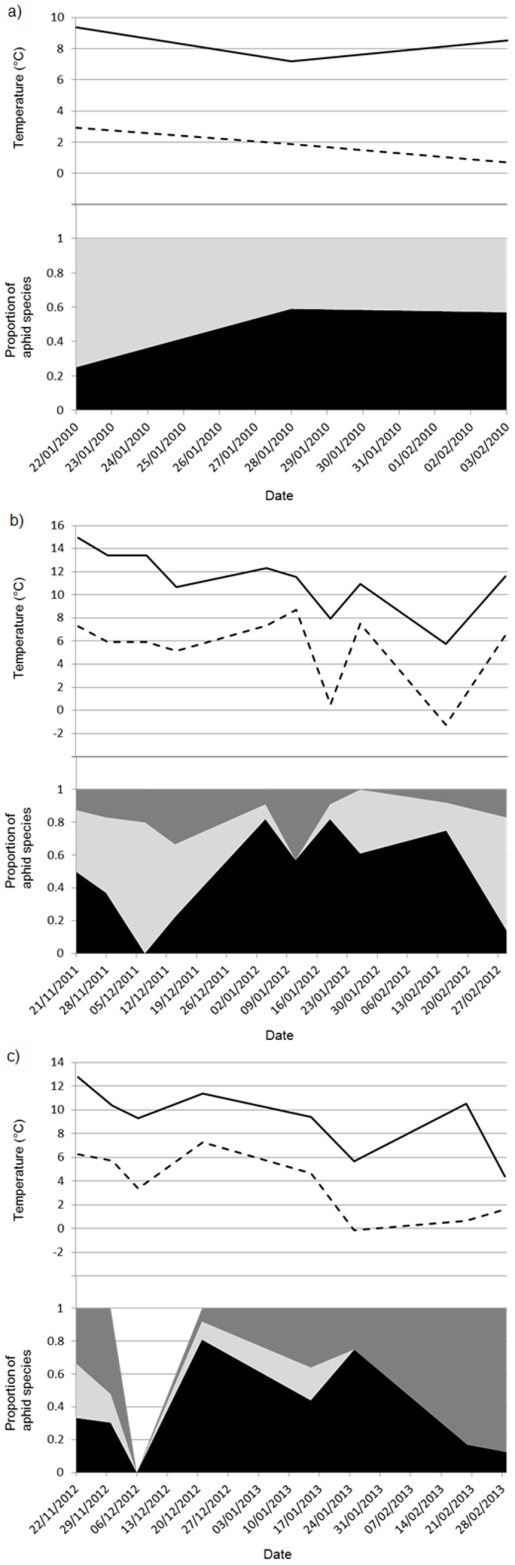
Species composition of the aphid population during winter sampling in fields of winter wheat, oat and triticale in north-western France during the winters of a) 2009/10, b) 2011/12, and c) 2012/13. *Metopolophium dirhodum* is indicated by the dark grey area, *Rhopalosiphum padi* by the black area and *Sitobion avenae* by the light grey area. Also shown is the average daily minimum (dashed line) and maximum (solid line) temperature (°C) 1 week prior to each sampling date.

Species composition of the aphid population in winters 2009/10, 2011/12 and 2012/13 are shown in Fig. 1. The combined totals of aphids collected on each sampling date used to calculate the proportions were as follows: a) Winter 2009/10∶22/01/2010 (n = 8), 28/01/2010 (n = 17), 03/02/2010 (n = 7); b) Winter 2011/12∶21/11/2011 (n = 8), 28/11/2011 (n = 24), 07/12/2011(n = 15), 14/12/2011 (n = 9), 04/01/2012 (n = 11), 11/01/2012 (n = 14), 19/01/2012 (n = 11), 26/01/2012 (n = 23), 15/02/2012 (n = 12), 29/02/2012 (n = 36); c) Winter 2012/13∶22/11/2012 (n = 3), 30/11/2012 (n = 23), 06/12/2012 (n = 0), 21/12/2012 (n = 62), 15/01/2013 (n = 25), 25/01/2013 (n = 4), 20/02/2013 (n = 12), 01/03/2013 (n = 8). No aphids were found in the winter of 2010/11. The mean daily minimum and maximum temperature one week prior to each sampling period is further displayed. Of the study winters, 2010/11 was the coldest and 2011/12 the warmest. In 2009/10, no *M. dirhodum* were found in the field and *S. avenae* and *R. padi* were present in almost equal numbers. For winters 2011/12 and 2012/13, *R. padi* constituted the greatest proportion of the aphid field population in winter months, with their numbers declining rapidly into spring. In the winter of 2011/12, *S. avenae* increased to dominance by late February. In contrast, in the winter of 2012/13, *M. dirhodum* increased to dominance by mid to late February.

### Lower lethal temperature (LT_50_)

*S. avenae* was significantly the most cold tolerant of the three aphid species regardless of aphid life stage, and *R. padi* the least cold tolerant, (Table 1). For *S. avenae* and *M. dirhodum*, adults were significantly more cold tolerant than first-instar nymphs. In contrast, for *R. padi*, the first-instar nymphs were significantly more cold tolerant that the adult stage (Table 1).

**Table 1:** Lower lethal temperatures (LT_50_) ±95% fiducial limit (°C) of first-instar nymphs and adults of the aphids *Sitobion avenae*, *Rhopalosiphum padi* and *Metopolophium dirhodum* acclimated at 20°C.

	Aphid life stage			
Species	First-instar nymph	*n*	Adult	*n*
				
*Sitobion avenae*	−7.5±0.2^e^	444	−8.4±0.2^f^	378
*Rhopalosiphum padi*	−4.9±0.3^b^	499	−3.6±0.5^a^	442
*Metopolophium dirhodum*	−5.8±0.3^c^	408	−6.6±0.3^d^	282

Different letters indicate significant differences in LT_50_, as determined by non-overlapping 95% fiducial limits.

### Aphid behaviour in a declining temperature regime

**Figure 2:**
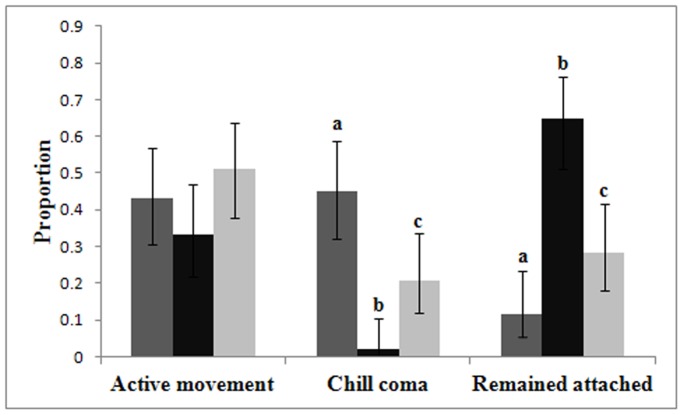
The relative proportion of behaviours exhibited by apterous adults of three aphid species (*Metopolophium dirhodum* (n = 51) indicated by the dark grey bars, *Rhopalosiphum padi* (n = 51) indicated by the black bars, and *Sitobion avenae* (n = 53) indicated by the light grey bars) when subjected to a declining temperature regime from 20°C to −10°C at 0.75°C min^-1^. Behaviours include: 1) the aphid actively left the host plant; 2) the aphid fell from the host plant as a result of chill coma; 3) the aphid remained attached to the host plant to temperatures of −10°C.

Significant differences in the behaviours exhibited by the three aphid species in a declining temperature regime were observed (χ^2^_4_ = 44.53, p<0.001) (Fig. 2). Species differences in the proportion of aphids that actively left the host plant were not observed (χ^2^_2_ = 2.27, p = 0.321), with approximate proportions for *M. dirhodum*, *R. padi* and *S. avenae* being 0.43, 0.33 and 0.51 respectively. Furthermore, significant differences in the temperature at which aphids of the three species actively moved from the host plant were not observed (χ22 = 3.47, p = 0.176).

**Figure 3:**
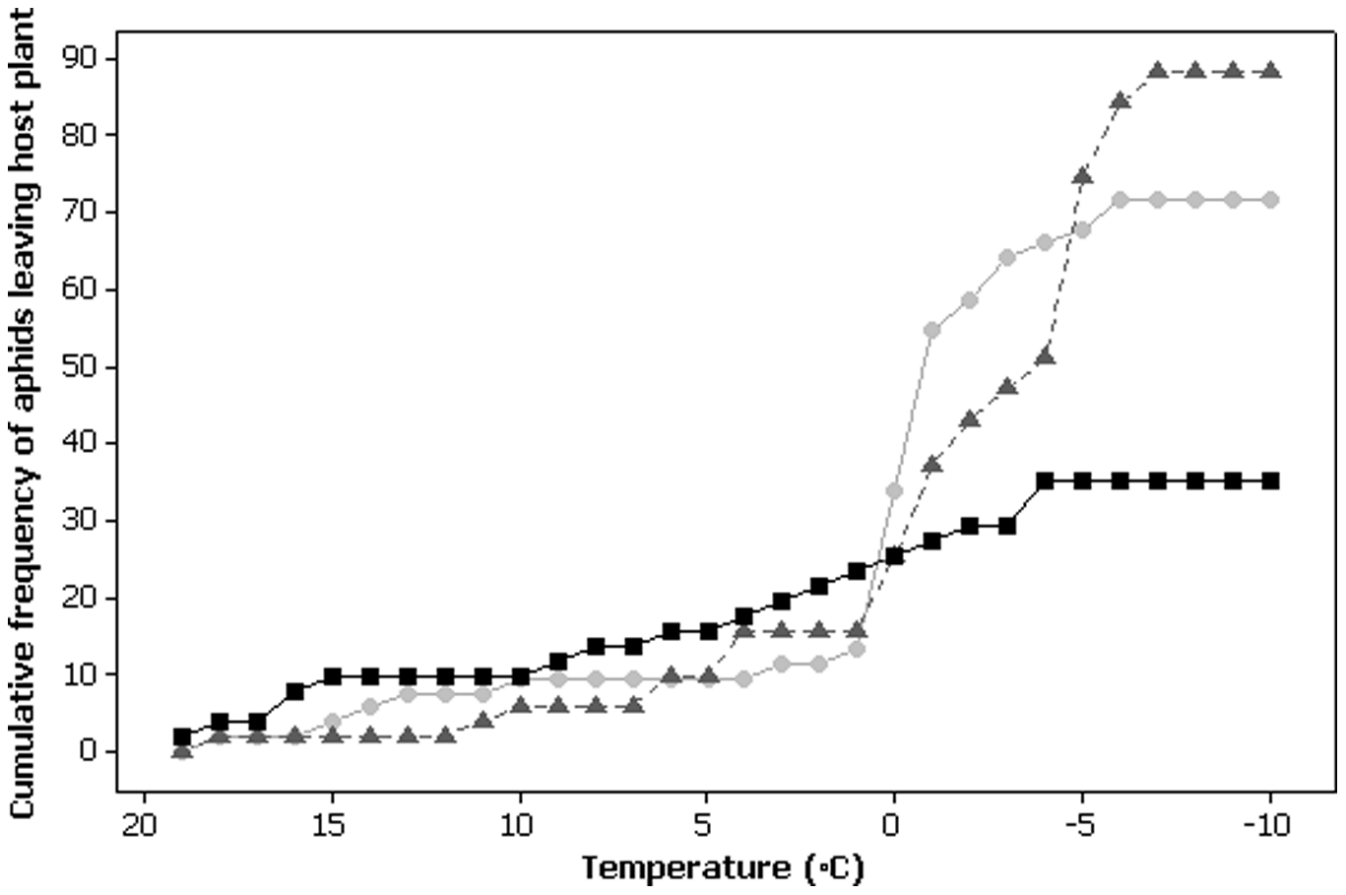
Cumulative frequency of apterous adult aphids of three species leaving the host plant (either through active movement or as a result of chill coma induction) when subjected to a declining temperature regime from 20°C to −10°C at 0.75°C min^-1^. *Metopolophium dirhodum* (dark grey triangles) (n = 51), *Rhopalosiphum padi* (black squares) (n = 51) and *Sitobion avenae* (light grey circles) (n = 53).

In contrast, species differences in the proportion of aphids that fell from the host plant as a result of chill coma were highly significant (χ^2^_2_ = 20.80 p<0.001), with significantly more *M. dirhodum* falling from the plant as a result of chill coma, and significantly less *R. padi* than expected (relative proportions for *M. dirhodum*, *R. padi* and *S. avenae* are 0.45, 0.02 and 0.21 respectively). Conversely, significantly more *R. padi* remained attached to the host plant for the duration of the declining temperature regime (i.e. until −10°C was reached), and significantly less *M. dirhodum* than expected (relative proportions for *M. dirhodum*, *R. padi* and *S. avenae* are 0.12, 0.65 and 0.28 respectively). Following cessation of the declining temperature regime, approximately 88% of *M. dirhodum* and 72% of *S. avenae* had become detached from the host plant, either through active movement from the plant or as a result of entering a chill coma causing the individual to fall from the plant (Fig. 3). In contrast, only 35% of *R. padi* were detached from the plant, proving significantly different from both *M. dirhodum* and *S. avenae* (χ^2^_1_ = 29.87 p<0.001).

Only one individual of *R. padi* fell from the host plant as a result of chill coma at a temperature of −3.7°C. Consequently *R. padi* were excluded from subsequent analyses. Significant differences in the chill coma temperature were observed between *S. avenae* and *M. dirhodum* at the culture temperature of 20°C (χ^2^_1_ = 7.36, p = 0.007). *M. dirhodum* dropped from the host plant at significantly lower temperatures (mean: −4.6±0.5°C) than *S. avenae* (mean: *−*2.7±1.2°C). Significant differences between scale parameters were observed (χ^2^_1_ = 4.01, p = 0.0045), with 95% of *M. dirhodum* individuals entering a chill coma rapidly over a temperature range of 1.0°C (between −4.1 and −5.1°C). In contrast, *S. avenae* entered chill coma over a more prolonged temperature range of 2.4°C (between −1.5 and −3.9°C).

## Discussion

Aphid abundance was recorded for four consecutive winters (2009/10 to 2012/13) in temperate cereal fields of north-western France. Of the years where aphids were successfully sampled, *R. padi* constituted the greatest proportion of the aphid population during the winter months, although their numbers declined rapidly into spring when either *S. avenae* or *M. dirhodum* increased to dominance. By mid spring, *S. avenae* dominates in the cereal fields of north-western France [56]. *R. padi* prefers cool, humid conditions, while *S. avenae*, in contrast, thrives in warm and dry conditions [57]–[59]. This variation in optimal conditions could explain the dominance of *R. padi* in the current study and the change in dominance to *S. avenae* by spring months [56]. Furthermore, winter temperatures in north-western France have increased in recent years leading to a rise in the winter abundance of *S. avenae* [48].

To better understand the mechanisms underlying differential winter abundance of aphid species, thermal tolerance and behavioural variations were investigated. Of the three aphid species common to temperate cereal fields, *R. padi* had the lowest physiological thermotolerance (LT_50_), dying at sub-zero temperatures significantly higher than its counterparts, and *S. avenae* the greatest physiological thermotolerance; a pattern supported by Williams [60]. Based on current abundance data, in conjunction with recent findings which suggest a negative correlation between *R. padi* relative abundance and temperature in temperate cereal fields [48], this result is perhaps counterintuitive. Furthermore, *S. avenae*, the most cold tolerant of the species, displayed a positive correlation between relative abundance and temperature [48]. In combination, this information suggests that variation in physiological thermotolerance does not contribute to the differential winter success of cereal aphids.

Long term meteorological data reveal that winter average daily minimum and maximum temperatures in north-western France have increased since the late 1980 s [48]. In conjunction with the data collected in the current study, meteorological data suggest that it is uncommon for aphids in temperate climates, such as north-western France, to experience temperatures below their lethal temperature. As such, the lethal temperature (i.e. intrinsic thermal tolerance) of an aphid may not be as important as once considered in predicting winter success, at least not over small spatial scales in temperate climates like those considered in the current study.

In addition to physiological thermotolerance, the current study investigated adult aphid behaviour in a declining temperature regime in the presence of host plant material to better understand how behaviour may impact aphid survival at low temperatures. Marked behavioural differences between the three species were observed. For *S. avenae*, the species with the greatest physiological thermotolerance, almost three quarters of individuals became detached from the host plant during the declining temperature regime. This contrasts greatly with *R. padi*, the least cold tolerant species, for which approximately one third of aphids became detached from the host, of which, only one individual fell as the result of a chill induced coma. Significantly more *S. avenae* and *M. dirhodum* fell from the plant as a result of chill coma than *R. padi*. Furthermore, *S. avenae* entered a chill coma at significantly higher temperatures than *M. dirhodum* (-2.7°C compared to −4.6°C). Therefore, patterns in physiological thermal tolerance did not match observed behavioural responses and chill coma induction.

Although the least cold tolerant species, it is possible that the success of *R. padi* in winter months could lie, not in its thermal tolerance, but instead in its ability to remain attached to the host plant down to extreme sub-zero temperatures. For example, contact with the host plant is known to enhance aphid cold tolerance [61], and could provide *R. padi* with a competitive advantage in winter months. Furthermore, by remaining attached to the host plant, *R. padi* avoids the costs associated with becoming detached from the host plant. Such detrimental effects of detachment from the host plant are well documented given that aphids frequently engage in a dropping behaviour to escape from enemy attack [62], [63]. Commonly incurred costs include lost feeding opportunity [64], desiccation-induced mortality [64], [65] and predation from ground predators should the aphid move from the host plant entirely [66]–[68]. Such costs are also likely to be experienced by aphids detached from the host plant as a result of cold temperatures. In remaining attached to the host plant, *R. padi* will avoid potential mortality associated with host detachment, further enhancing its competitive advantage over its counterparts.

Given the variation in behaviour of the three species, in conjunction with information on aphid winter abundance, behaviour could play a vital role in shaping aphid winter populations. However, it is worth noting that, at low temperature extremes, the probability of survival is dependent upon the interaction between temperature and duration of exposure [69]. Subsequently, the observed mortality and behavioural responses in the current study could occur at higher temperatures if the duration of exposure is extended. Longer exposures at higher temperatures, such as those likely in the field, could see aphids such as *S. avenae* and *M. dirhodum* (i.e. species which display a higher proportion of host detachment at low temperatures) increasingly vulnerable to the associated costs of host detachment (lost feeding opportunity, desiccation and ground predation) at higher temperatures than those observed in the current study.

The findings of this study illustrate the limitations to studying physiological thermotolerance in isolation under laboratory conditions. Based solely on physiological thermotolerance, it would be incorrectly concluded that *R. padi* is the least adapted to winter conditions of the cereal aphids. Instead, recorded field abundances of cereal aphids confirm the converse (see also [48]–[50], [70]). However, investigation into the relative behaviours of the aphid species at unfavourable cold temperatures revealed behavioural variations that could instead contribute to the differential winter survival observed. The inclusion of behavioural thermoregulation traits into studies of insect thermotolerance therefore has the potential to greatly enhance our understanding of insect, and indeed ectotherm, survival at extreme temperatures, particularly in temperate climates where lower lethal limits are rarely reached and may subsequently play little role in shaping winter populations. With a rise in the number of studies on insect thermal tolerance, particularly in predicting impacts to species under climate change scenarios, these results are particularly pertinent since physiological thermotolerance measures in isolation could lead to misleading or inaccurate conclusions.

Furthermore, recent work suggests that most ectotherms do not possess a physiological thermal safety margin as once thought [32]. As such, ectotherms will be forced to rely increasingly on behavioural thermoregulation to avoid and survive extreme climatic events and climate warming [32]. Understanding how ectotherms utilise their environment to avoid unfavourable thermal conditions is not only fundamental to our understanding of species’ vulnerability to climate change and extreme events, but imperative if we are to manage landscapes in a way to provide ectotherms with the thermal refuges they require to survive and persist.
